# Bridging the gap: enhancing blood regulatory functions in African contexts through comparative analysis

**DOI:** 10.3389/fmed.2025.1519719

**Published:** 2025-05-01

**Authors:** Washington T. Samukange, Verena Kluempers, Chancelar Kafere, Kristina Heinrich, Joanna Atemnkeng, Alireza Khadem Broojerdi, Florence Tirane, Edwin Nkansah, Shani Maboko, Linda Nhukarume, Khamusi Mutoti, Noel Aineplan, Helga Gardasdottir, Aukje K. Mantel-Teeuwisse, Jens Reinhardt

**Affiliations:** ^1^Centre for International Cooperation, Paul-Ehrlich-Institut, Langen, Germany; ^2^Division of Pharmacoepidemiology & Clinical Pharmacology, Faculty of Science, Utrecht Institute for Pharmaceutical Sciences (UIPS), Utrecht University, Utrecht, Netherlands; ^3^Expertise and Think, Enabel, Belgian Development Agency, Brussels, Belgium; ^4^World Health Organisation, Regulatory Systems Strengthening, Regulation and Safety Unit, Geneva, Switzerland; ^5^Zambia Medicines Regulatory Authority, Lusaka, Zambia; ^6^Food and Drugs Authority Ghana, Accra, Ghana; ^7^Tanzania Medicine and Medical Devices Authority, Dodoma, Tanzania; ^8^Medicines Control Authority of Zimbabwe (MCAZ), Harare, Zimbabwe; ^9^South African Health Products Regulatory Authority (SAHPRA), Pretoria, South Africa; ^10^National Drug Authority (NDA), Kampala, Uganda; ^11^Department of Clinical Pharmacy, University Medical Center Utrecht, Utrecht, Netherlands; ^12^Department of Pharmaceutical Sciences, University of Iceland, Reykjavik, Iceland; ^13^Division of Haematology, Cell and Gene Therapy, Paul-Ehrlich-Institut, Langen, Germany

**Keywords:** blood and blood products, global benchmarking tool, Sub-Saharan African countries, availability of safe blood, GHPP BloodTrain

## Abstract

**Introduction:**

Independent assessments of blood regulatory systems, facilitated by tools such as the WHO's Global Benchmarking Tool (GBT) plus Blood expedites development of National Regulatory Authorites (NRAs) and thus promotes increased access to safe, effective, and quality blood, blood components, and products. The aim of this study was to assess and compare the status of implementation and performance of the regulatory functions for registration and marketing authorization as well as the system for approval of blood, blood components and plasma for fractionation or processes.

**Methods:**

We did this by conducting assisted self-benchmarking in 12 African countries using the GBT plus Blood (registration and marketing authorization function, 34 sub-indicators and approval of blood, blood components, and plasma for fractionation or processes function, 24 sub-indicators). Comparative assessments of WHO-designated maturity level 3 (ML3) NRAs for medicines and vaccines against non-designated NRAs were made.

**Results:**

The percentage of implemented sub-indicators was higher for the registration and marketing authorization function with an average implementation score of 73% (range: 51%−92%) compared to the approval of blood, blood components, and plasma for fractionation or processes function which had an average implementation score of 45% (range: 6%−65%). The comparison of group averages for the ML3-designated NRAs against the non-designated NRAs revealed a higher score 91% (range: 71%−100%) for ML3-designated NRAs as opposed to a lower score of 71% (range: 49%−100%) for the non-designated NRAs for the registration and marketing authorization function. This pattern, however, was not observed for the comparison of group averages for the approval of blood, blood components, and plasma for fractionation or processes function where the ML3-designated NRAs scored 47% (range 19%−72%) against 46% (range 23%−88%) for the non-ML3-designated NRAs.

**Conclusion:**

Most of the NRAs excelled in implementing sub-indicators for the registration and marketing authorization (of plasma-derived medicines) function. All NRAs exhibited notable flaws in regulating blood, blood components, plasma for fraction, and approval of processes, indicating nascent regulatory frameworks. This study highlights the urgent need for WHO and African countries to prioritize formal benchmarking of NRAs using the GBT plus Blood to enhance their regulatory capacities in blood and blood product regulation.

## Introduction

The need for blood regulation arises from the inherent dangers of blood and blood products, and the complexities of preparation of whole blood and blood components for transfusion and the manufacture of plasma-derived medicinal products (1–3). Threats to blood quality and blood safety from different viruses and from newly emerging blood-borne diseases have resulted in increased blood quality and safety concerns (4). In Africa, major concerns remain over safety risks posed by high rates of transfusion transmissible infectious diseases in the general population (4–6).

Recognising blood and blood products as essential medicines highlight their crucial importance in healthcare systems (7, 8). Every country needs to have an assured supply of safe, efficacious, good quality and affordable blood, and blood products to promote public health and patient care (9, 10). The lack of effective blood regulatory systems can thus be a barrier in access to blood, blood components, and blood products (11). The need to ensure “appropriate regulatory systems” in the area of quality and safety of blood and blood products was recognized in the 2010 World Health Assembly resolution 63.12 (12). Robust and effective blood regulation therefore plays a vital link between improving equitable access to blood and blood products, promoting adequacy of blood supply and assuring blood quality.

Competent national regulatory authorities (NRAs) have the mandate to ensure consistent compliance with appropriate quality and safety standards for blood and blood products. This is achieved through a set of regulatory control measures such as registration and marketing authorization of plasma-derived medicines and approval of blood, blood components, including plasma for fractionation (concerning the product and/or the manufacturing process) among others (3, 13). The former pertains to the mechanism for issuance of marketing authorizations, or registrations, of plasma derived medicines subsequent to an evaluative procedure assessing their quality, safety, and efficacy (14–16). The approval of blood, blood components, and plasma for fractionation involves a regulatory mechanism ensuring the adherence to established standards for quality, safety, and efficacy, as well as the suitability of product information pertaining to blood and its components, including plasma for fractionation, or the processes involved in their preparation (14–16). NRAs play an integral role in national blood systems destined to ensure equitable access to essential blood and blood products of assured quality, safety, and efficacy (17).

Independent assessment of blood-related regulatory functions (such as those detailed above) and their implementation in a country has the potential to bolster confidence in regulatory competence. Moreover, such assessments have the capacity to catalyse the augmentation of NRA competencies in blood regulation and ultimately improve access to safe, effective, and quality blood and blood products (3, 13). The WHO has included blood and blood product regulation in its Global Benchmarking Tool (GBT) for the evaluation of national regulatory systems for blood and blood products (14–16). The GBT plus Blood is used to assess and measure the performance of each regulatory function, that is the national regulatory system, registration and marketing authorization, vigilance (haemovigilance), licensing of blood establishments, market control and post-marketing surveillance, regulatory inspections, clinical trial authorization, lot release and lab access, approval of blood and blood components including plasma for fractionation (or processes involved in their preparation), and approval of medical devices and associated substances and *in-vitro* diagnostic (IVD) and medical devices (14, 15). The evaluation assesses competencies and maturity of blood regulation at the NRA and identifies deficiencies as a basis for continuous improvement (10).

WHO estimates that only 30% of NRAs have adequate capacity to perform the core regulatory functions for medicines and vaccines globally (18, 19). Further, only 37% of countries in Africa reported having a system for authorization and/or approval of blood establishments as well as licensing of blood establishments in the WHO Global Status Report on Blood Safety and Availability (2018) (11). Detailed information on the performance of countries in blood regulatory functions, however, is lacking. In the meantime, WHO has designated only 8 countries (Egypt, Ghana, Nigeria, Tanzania, Rwanda, Senegal, South Africa, and Zimbabwe) in Africa to be operating at maturity level 3 (ML3), that is having the minimal capabilities of a stable, well-functioning and integrated regulatory system to meet local needs. Of these, only Egypt is ML3 for both medicines and imported vaccines (non-producing) and also ML3 for local vaccines (producing). Ghana, Nigeria, Tanzania, Rwanda, Senegal, and Zimbabwe are only ML3 for medicines and imported vaccines. South Africa is ML3 only for local vaccines (producing).

The aim of this study was to assess and compare the status of implementation and performance of the system for registration and marketing authorization as well as the system for approval of blood, blood components, and plasma for fractionation or the respective manufacturing processes in Africa. We did this by conducting assisted self-benchmarking assessments in 12 countries using the WHO GBT plus Blood over a 5-year period. Further, we compared the implementation and performance of countries that are already deemed to be operating at maturity level 3 (ML3) for medicines and vaccines (non-producing) and/or deemed to be operating at ML3 for local vaccines (producing) against those yet to achieve this status. These comparisons provide vast potential for within and cross-country learning by offering a way to explore different approaches countries take to address similar problems to achieve comparable objectives.

## Methods

### Study design

This cross-sectional descriptive study examined the existing systems for the registration and marketing authorization of plasma-derived medicines, as well as the approval processes for blood, blood components, and plasma for fractionation in 12 Sub-Saharan African countries: Ghana, Ethiopia, Nigeria, Malawi, Kenya, Liberia, Rwanda, Uganda, South Africa, Tanzania, Zambia, and Zimbabwe. Table 1 provides an overview of the National Regulatory Authorities (NRAs) and the blood regulatory systems that were benchmarked, along with the dates when the data from these systems were updated. The self-benchmarking process is detailed in the WHO Global Benchmarking Tool (GBT) for evaluating national regulatory systems for medical products, and the “Manual for Benchmarking and Formulation of Institutional Development Plans” (20).

**Table 1:** Benchmarking and updates of national blood regulatory systems across 12 countries.

**Country**	**National Regulatory Authority**	**Blood Collection and Supply Institution**	**Initial benchmarking**	**Blood regulatory systems**		
			**2018**	**2021**	**2022**	**2023**
Ethiopia	Ethiopian Food and Drug Administration (EFDA)	National Blood Bank Service and Red Cross Society	✓	✗	✓	✗
Ghana	Food and Drugs Authority Ghana	National Blood Service Ghana	✓	✓	✓	✓
Kenya	Pharmacy and Poisons Board (PPB)	Kenya National Blood Transfusion Service	✓	✓	✓	✗
Liberia	Liberia Medicines and Healthcare Products Regulatory Authority (LMHRA)	Blood Safety Program, Ministry of Health	✓	✗	✓	✗
Malawi	Pharmacy Medicines and Poisons Board of Malawi (PMPB)	Malawi National Blood Transfusion Service	✓	✗	✓	✗
Nigeria	National Agency for Food and Drug Administration and Control	National Blood Transfusion Service of Nigeria, Regional and State Blood Transfusion Services	✓	✓	✓	✗
Rwanda	Rwanda Food and Drugs Authority (RFDA)	National Centre for Blood Transfusion	✓	✗	✓	✗
South Africa	South African Health Products Authority (SAHPRA)	South African National Blood Service and Western Cape Blood Service	✓	✓	✓	✓
Tanzania	Tanzania Medicines and Medical Devices Authority (TMDA)	National Blood Transfusion Service Tanzania and Regional Hospitals	✓	✓	✓	✓
Uganda	National Drug Authority (NDA)	Uganda Blood Transfusion Services	✓	✗	✓	✗
Zambia	Zambia Medicines Regulatory Authority	Zambia National Blood Transfusion Service	✓	✓	✓	✓
Zimbabwe	Medicines Control Authority of Zimbabwe (MCAZ)	National Blood Services Zimbabwe	✓	✓	✓	✓

### Indicators and sub-indicators

The GBT plus Blood employs a comprehensive set of 14 indicators which are utilized for the registration and marketing authorization (*n* = 6), as well as the approval of blood, blood components, and plasma for fractionation or process functions (*n* = 8). These indicators are further divided into 58 sub-indicators to comprehensively compare, evaluate, and measure the performance and implementation of these two blood regulatory functions. For the registration and marketing authorization function, six indicators are used, each covering the following specific themes (1) legal provisions, regulations and guidelines, (2) organisation and governance, (3) human resources, (4) regulatory processes, (5) transparency and accountability, and (6) monitoring progress and assessing impact. Conversely, the approval of blood, blood components and plasma for fractionation or processes function was evaluated using eight indicators, each covering a specific theme: (1) legal provisions, (2) system to ensure quality, safety, and efficacy of blood and blood components, (3) criteria for donor selection and deferral, (4) requirements for transmissible-disease testing, (5) requirements for labelling, (6) approval system for blood and blood components, (7) requirements for post-approval changes, and (8) existence of appropriate expertise. This structured approach ensures consistency in the GBT plus Blood tool (15, 20). Furthermore, the GBT plus Blood tool comprises both common sub-indicators (applicable to medicines, vaccines, and blood and blood products) and specific (non-common) sub-indicators (blood and blood product specific sub-indicators). For the function of registration and marketing authorization, there are non-common sub-indicators (*n* = 3) and common sub-indicators (*n* = 31), while the approval of blood, blood components, and plasma for fractionation or processes utilizes only non-common sub-indicators (*n* = 24).

The WHO GBT also incorporates the maturity level concept from the International Standard Organisation (ISO) 9004:2018 (15, 19). This concept enables the assessment of the status and performance of regulation with a variety of indicators and sub-indicators and gives an overall view of the NRA's maturity based on the achievement of general benchmarks in regulatory practice. The maturity levels for the sub-indicators are distributed as shown in Supplementary Tables 1a, b.

While in some of the countries, sub-indicators for ML4 were also assessed, this was not done in all areas, therefore not allowing a general analysis. Therefore, the results are not included in this publication.

### Benchmarking methodology and data collection

Before visiting the participating NRAs, authorization and approval for the benchmarking was sought from the heads of agencies via e-mail in 2018. Key individuals with overall responsibility and knowledge of the respective national system in each country were identified. They were informed about the assessment and asked to share the legal and statutory documents and other relevant information with the external assessment team before the benchmarking visit. The documents requested were extracts of national legislation describing responsibilities of the function of the registration and marketing authorization and/or the systems for approval of blood, blood components, and plasma for fraction, regulations, and guidelines.

A priori data from the previous self-benchmarking by the NRA was also sent to the Paul-Ehrlich-Institut (PEI) BloodTrain team, where it was available and was used to pre-fill the sub-indicators before each visit. The actual assisted self-assessment was carried out on-site at each of the NRA's premises with the NRA's team as an assisted self-benchmarking exercise to complete the WHO GBT plus Blood. The initial self-assessments and data collections in the 12 countries were conducted from 2018. Further, updated data was collected from each NRA, where specific updates and changes were available from in July 2021 and in April 2022 and validated by the same team and where there were updates these were noted (See Table 1).

The benchmarking principles on assessment procedures and conducting benchmarking assessments (how to score, evidence to review) that are enshrined in the WHO Manual for benchmarking of the national regulatory system of medical products were applied (20). The GBT plus Blood was completed by senior staff from the registration and marketing authorization of blood products and approval of blood (product/process), blood components, and plasma for fractionation teams of the national regulatory agencies with the support of the BloodTrain team. Data were collected and recorded in the data collection module of the WHO GBT application and validated by the BloodTrain team.

To determine whether a sub-indicator was implemented or not, the NRA had to provide documentary evidence and references. When documentary evidence such as legislation (Act or Regulation), policy, and/or guidelines that were being implemented and enforced were available, the sub-indicator would be scored “Implemented” and the system would give a numerical score of 1. When the NRA had documentary evidence (such as legislative provisions, policy, guidelines or procedures) without any further evidence of implementation or was still at the initial stages of implementation of their legal requirements, the sub-indicator was scored “partially implemented” and the system scored the sub-indicator with a score of 0.75 (20). When the NRA had recently drafted legislation or guidelines that were not being followed, the sub-indicator was scored “ongoing implementation” and the system would give a numerical score of 0.25. When the NRA was not implementing the sub-indicator or had neither documentary evidence nor references to satisfy the requirement of the sub-indicator, then the sub-indicator was scored “not implemented” and the system would give this a numerical score of 0.

While in some NRAs the indicators up to ML4 were assessed during the piloting of the tool, this was not done systematically. Therefore, the focus of the data analysis for the 12 African NRAs was up to ML3.

### Data analysis

The data from each country‘s self-assessment were collected in the WHO GBT plus Blood data collection module and exported into a Microsoft Excel (Microsoft Corporation, Redmond, WA, USA) template from the WHO GBT plus Blood with all indicators.

To determine the status of implementation of registration and marketing authorization and approval of blood and blood components sub-indicators in each country, the sum of the sub-indicator scores were expressed as a percentage of the maximum score that could be obtained. Similarly, to determine the performance of specific registration and marketing authorization and approval of blood and blood components functions, the sum of sub-indicator responses for each indicator was analysed. The maturity levels of the two regulatory functions of each country were analysed by comparing the sum of the responses to each of the sub-indicators against their maturity levels. Additionally, we compared the implementation and performance of WHO-designated ML3 NRAs for medicines or vaccines (producing or non-producing) against those that are not WHO-designated ML3 NRAs by averaging the groups and expressing this as percentage. The authorities were anonymized randomly for the presentation of results.

## Results

**Figure 1:**
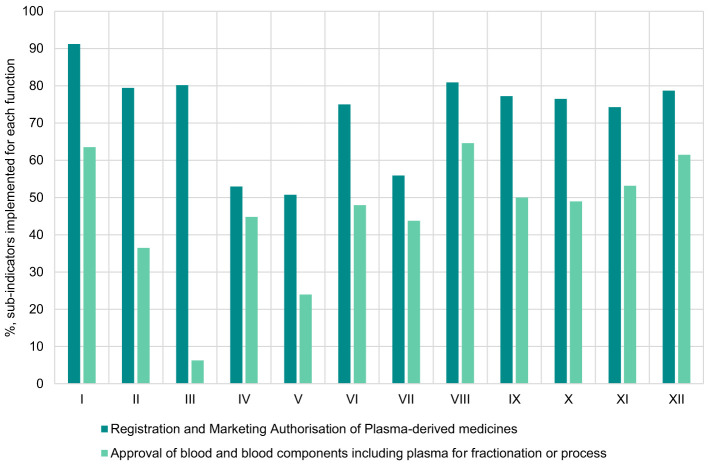
Overall implementation of registration and marketing authorisation of plasma-derived medicines and approval of blood and blood components including plasma for fractionation or processes functions in benchmarked country NRAs.

Overall, a greater number of sub-indicators were implemented for the registration and marketing authorization function compared to the approval of blood and blood components or processes function. For the registration and marketing authorization function, the average score for implementation of sub-indicators was 73% (range: 51%−92%), with eight countries achieving a score of at least 80% (Figure 1). In contrast, for the approval of blood and blood components including plasma for fractionation function, the average score for implementation was 45% (range: 6%−65%) with five countries having an implementation score of at least 50%. We also noted that, for the two functions, none of the NRAs scored 100% implementation of all sub-indicators and as such none of the NRAs were operating at ML3 level for the 2 functions for blood regulation.

**Figure 2:**
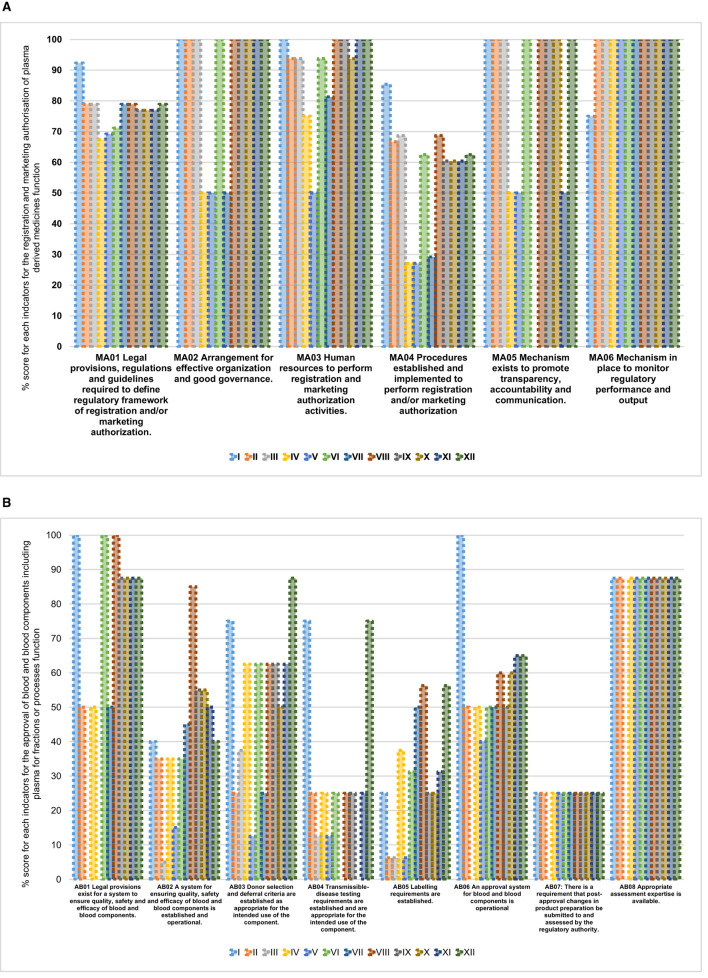
**(A)** Overall performance of registration and marketing authorization of plasma-derived medicines function in benchmarked country NRAs. **(B)** Overall performance of approval of blood and blood components including plasma for fractionation or processes function in benchmarked country NRAs

All NRAs had legislative provisions mandating them to perform the registration and marketing authorization function with all NRAs implementing on average 70% (range: 67%−92%) for this indicator (Figure 2A). The procedures to perform registration and marketing authorization were the least implemented indicator among the 12 NRAs with an average implementation score of 57% (range: 27%−85%). The rest of the indicators for the registration and marketing authorization function had good implementation scores. For the same function, we also observed that the non-common sub-indicators were not fully implemented in any of the NRAs.

Among the eight indicators for approval of blood and blood components or processes function, the highest average implementation scores were observed for the following indicators: availability of appropriate assessment expertise 80% (range: 0%−88%), existence of legal provisions for systems to ensure quality, safety, and efficacy of blood and blood components 67% (range: 0%−100%), approval system for blood and blood components is in place 53% (range: 0%−100%) and donor selection and deferral criteria are established 52% (range: 13%−88%) (Figure 2B). The remaining indicators had average implementation scores below 50%.

**Figure 3:**
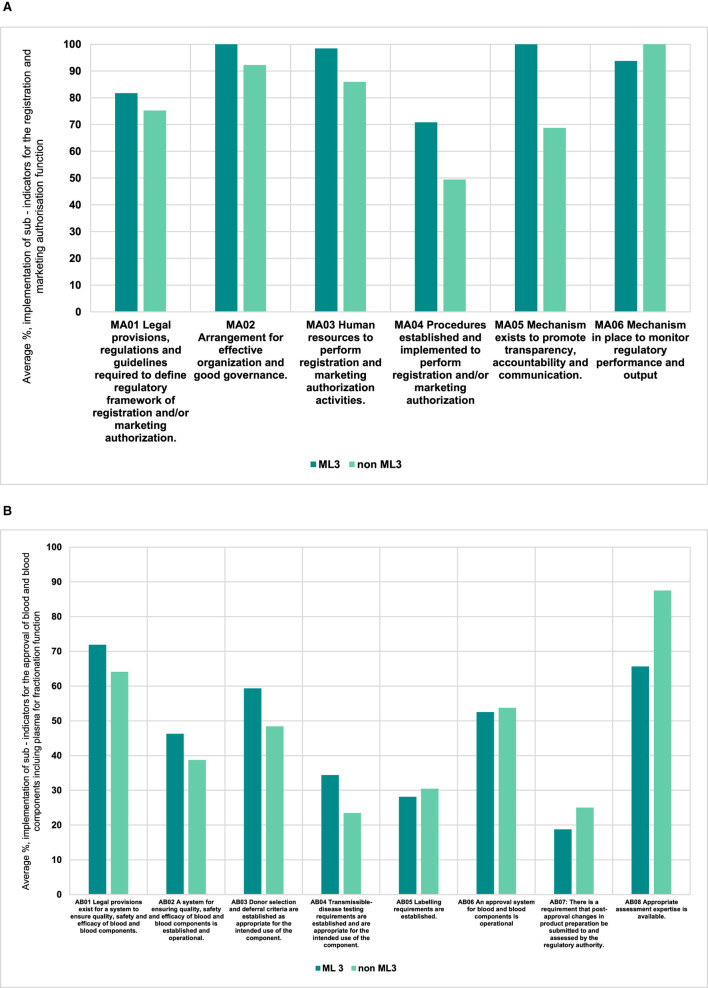
**(A)** Comparison of performance of indicators for ML3 NRAs vs non ML3 countries for the registration and marketing authorization function (plasma — derived medicines). **(B)** Comparison of performance of indicators for ML3 NRAs vs non ML3 countries for the approval of blood and blood components including plasma for fractionation function.

We noted that those NRAs that operated already on ML3 for medicines and vaccines (producing or non-producing) had a higher average implementation score, which was 91% (range 71%−100%), than those that were not for the registration and marketing authorization (of plasma-derived medicines) function (Figure 3A). Further, the implementation scores were lower for the approval of blood and blood components or processes function both for those NRAs already WHO-designated ML3 (47% range 19%−72%) and those that were not (46% range 23%−88%) (Figure 3B). We further noted that for four indicators of the same function the non-WHO designated ML3 NRAs had the same average implementation scores or better than those of WHO designated ML3 countries.

## Discussion

Our study showed good implementation and performance of the registration and marketing authorization (of plasma-derived medicines) function in 9 of the 12 NRAs with scores above 72%, based on the WHO defined scoring system indicated in the WHO Manual for benchmarking of the national regulatory system of medical products and formulation of institutional development plans (20). However, the approval of blood, blood components, and plasma for fractionation or processes function demonstrated considerable deficiencies in its implementation and performance. We further noted substantial flaws in implementing specific (sub-indicators relating to blood and blood products in both functions. Our findings also showed the approval of blood, blood components, and plasma for fractionation or processes function had lower implementation scores compared to the registration and marketing authorization (of plasma-derived medicines) function. Additionally, the implementation and performance of the approval of blood, blood components, and plasma for fractionation or processes function was comparable between NRAs at ML3 and other NRAs. Notably, no NRAs achieved the stable, well-functioning, and integrated system or maturity level 3 rating for blood and blood products. These insights can be integrated into existing efforts to enhance blood regulation in African countries (13).

In our comparison of the two blood regulatory functions, we observed that 9 out 12 NRAs demonstrated high implementation of sub-indicators (with scores above 72%) for the registration and marketing authorization (of plasma-derived medicines). The implementation of sub-indicators for the approval of blood, blood components, and plasma for fractionation or processes function showed significant shortcomings, with only four NRAs achieving implementation scores above 50%. This observation aligns with the global trend in the development of blood and blood product regulation, where stringent regulation for plasma-derived medicines followed immediately after the tragic scandals of transfusion-transmitted AIDS infections by blood transfusions and plasma derivatives (5, 21, 22). Plasma-derived medicines became subject to pharmaceutical legislation in Europe since 1989 and are similarly regulated in all 12 NRAs and the regulatory function is therefore well-implemented (22–25). Blood and blood components are subject to the blood directive (Directive 2002/98/EC) which has been transposed into national law in all EU states since 2002, much later than the establishment of regulation for plasma-derived medicines (23, 24, 26, 27). Comparable to Europe, other countries such as Australia, Canada, Japan, Singapore, South Korea, and Switzerland also saw the evolution of stringent regulation for blood and blood components occurring later than that for plasma-derived medicines (28).

Despite most NRAs in our study attaining high scores for implementing the registration and marketing authorization function, they fell short of fully implementing the three specific sub-indicators associated with blood and blood products. Notably, the common sub-indicators for medicines and vaccines were consistently well-implemented and NRAs with WHO designated ML3 status demonstrated effective implementation of these common sub-indicators (13, 20, 29, 30). WHO-designated ML3 NRAs and other NRAs in our study have invested effort in strengthening this function, and benefitted from the focused approach to build capacities and strengthen systems that WHO and other development partners have taken to improve the registration and marketing authorization function for medicines and vaccines (29, 31).

We found that most NRAs scored low in implementing the specific function “approval of blood, blood components, and plasma for fractionation or processes.” Even the more mature NRAs in our study were struggling in implementing the specific sub-indicators related to blood and blood products only. Coupled with the lack of full implementation of the non-common sub-indicators for the registration and marketing authorization function, it is evident that there are significant challenges for African NRAs in implementation of blood regulation, impacting the provision of safe, quality-assured blood and blood components (5, 6, 32). Of note is that independent national blood systems in African countries have developed with less stringent regulatory oversight, a similar observation reported in the EU as well (2, 22). Overall, the results we observed are synonymous with regulatory systems that are still in their early phases of implementation (22, 24, 25). Moreover, there are limited global initiatives to support low-and-middle income countries in enhancing their blood regulatory functions and capacity building. Most notable among the few current international opportunities to support blood and blood regulation in developing countries is the Bundesministerium für Gesundheit (Federal Ministry of Health, Germany) financed Global Health Protection Programme (GHPP)'s BloodTrain project that is implemented by the Paul-Ehrlich-Institut (9, 10, 33–36). The recently ended WHO Action Framework to Advance Universal Access to Safe, Effective and Quality-Assured Blood and Blood Products (2020–2023) was another renewed effort from WHO to among other things strengthen blood regulation (30).

Throughout our study, we came across concrete examples of common problems and challenges in implementing requirements of the WHO GBT plus Blood for the two functions across all NRAs which have been repeatedly reported for medicines and vaccines regulation. We identified four primary issues in our study as major challenges for blood regulation coming from both regulatory functions include: (1) legal provisions (system for ensuring quality, safety, and efficacy of blood and blood components), (2) selection, deferral and transmissible-disease testing requirements for blood, blood components, plasma for transfusion, and plasma for fractionation (6), (3) human resources (37–39), and (4) regulatory processes (38–42).

To improve the implementation and performance of the two functions, particularly the blood and blood product related requirements, sustained political will is necessary to prioritise national blood regulatory systems and national blood systems as the anchors of improving the access to quality-assured blood and blood products. It is imperative to further strengthen legislative measures to establish legal requirements for selection, deferral and transmissible-disease testing for blood, blood components, plasma for transfusion, and plasma for fractionation. It is essential to offer regular in-service opportunities (on the job training, mentoring, internships, joint assessments or supported assessments, workshops) and “twinning opportunities” with competent NRAs to provide continuous professional development, competence (43, 44), and capacity building opportunities for staff and NRAs in this crucial area of healthcare.

To foster collaboration and reliance, it is essential to accelerate global and regional regulatory harmonization and to enhance the overall efficiency in NRAs in Africa, e.g., by relying on WHO-designated ML3 NRAs (45). At the level of the African Union, the African Union Development Agency-Africa Medicines Regulatory Harmonisation (AMRH) programme has included strengthening of blood and blood product regulation through the African Blood Regulators Forum (ABRF)—continental technical working group. The focus of the African Medicines Agency (AMA) does not extend to blood and blood components but will include innovative plasma-derived medicines such as coagulation factors or recombinant analogues (46, 47). To sustainably build capacities in African NRAs, there is urgent need to designate Regional Centres of Regulatory Excellence (RCOREs) specifically dedicated to support blood regulation or any of the core functions of blood regulation. Capacitating and designating one would represent a crucial next step in the current efforts to strengthen blood regulation in Africa.

Benchmarking tools must be carefully designed and implemented to generate meaningful results. However, generating meaningful benchmarking data and properly evaluating performance in this complex domain remains challenging. In this study, the GBT + Blood did not measure regulatory outcomes such as the numbers of approved blood and blood products, timelines for approval or other key performance indicators. Further, while the information gathered was correct at the time of data collection, some NRAs may have updated their systems. Further studies about the positive effects of benchmarking or benchmarking outcomes are warranted to engage continuous commitment into the practice.

## Conclusion

This study contributes to our overall understanding of core elements of regulation of blood and blood products and provides insights into how the registration and marketing authorization (of plasma-derived medicines) function is well-implemented in all NRAs. However, the implementation of the approval of blood, blood components and plasma for fractionation reflected a system early in its infancy. Insights from our study can be utilized to expand knowledge on how to enhance blood regulatory systems to increase access to quality-assured blood and blood products. Benchmarking of NRAs with the WHO GBT plus Blood is essential for strengthening blood regulatory systems in Africa. It fosters performance comparisons, maturity level assignments, and targeted WHO advocacy for NRA support. Moreover, the AMRH programme's RCORE concept, with adequate financial resources, can serve as a vital element to enhance regulatory capacities across the continent.

## Data Availability

The datasets generated and/or analysed during the current study are not publicly available due to the confidential nature of the data, but are available from the corresponding author on reasonable request'. Requests to access the datasets should be directed to BloodTrain@pei.de.

## References

[1] Klein HG Hrouda JC Epstein JS. Crisis in the sustainability of the U.S. blood system. N Engl J Med. (2017) 377:1485–88. 10.1056/NEJMsb1706496 29020590

[2] Epstein J Seitz R Dhingra N Ganz PR Gharehbaghian A Spindel R. Role of regulatory agencies. Biologicals. (2009) 37:94–102. Available online at: https://www.sciencedirect.com/science/article/abs/pii/S1045105609000050?via%3Dihub 19230707 10.1016/j.biologicals.2009.01.004

[3] Epstein JS. Best practices in regulation of blood and blood products. Biologicals. 40:200–4. 10.1016/j.biologicals.2011.11.002 22122986

[4] Weimer A Tagny CT Tapko JB Gouws C Tobian AAR Ness PM. Blood transfusion safety in sub-Saharan Africa: a literature review of changes and challenges in the 21st century. Transfusion. (2019) 59:412–27. 10.1111/trf.14949 30615810

[5] Barro L Drew VJ Poda GG Tagny CT El-Ekiaby M Owusu-Ofori S. Blood transfusion in sub-Saharan Africa: understanding the missing gap and responding to present and future challenges. Vox Sang. (2018) 113:726–36. 10.1111/vox.12705 30221365

[6] Custer B Zou S Glynn SA Makani J Tagny CT Ekiaby M. Addressing gaps in international blood availability and transfusion safety in low- and middle-income countries: a NHLBI workshop. Transfusion. (2018) 58:1307–17. 10.1111/trf.14598 29542130 PMC6510980

[7] World Health Organization. WHO model list of essential medicines (2019). Available online at: https://www.who.int/publications/i/item/WHO-MHP-HPS-EML-2023.02 (accessed March 29, 2023).

[8] Samukange WT Gardarsdottir H Leufkens HGM Mantel-Teeuwisse AK. Selection of blood, blood components, and blood products as essential medicines in 105 low- and middle-income countries. Transfus Med Rev. (2020) 34:94–100. 10.1016/j.tmrv.2019.10.005 31761652

[9] Samukange WT Kafere C Heinrich K Sabblah GT Siame MM Chirinda L. Strengthening blood regulatory systems to tackle Africa's unmet needs for blood and blood products. Transfus Med Hemother. (2022) 50:123–8. 10.1159/000528077 37066057 PMC10090969

[10] Samukange WT Kluempers V Porwal M Mudyiwenyama L Mutoti K Aineplan N. Implementation and performance of haemovigilance systems in 10 sub-saharan African countries is sub-optimal. BMC Health Serv Res. (2021) 21:1258. 10.1186/s12913-021-07235-0 34801022 PMC8605544

[11] World Health Organization. Global status report on blood safety and availability 2021 (2022). Available online at: https://www.who.int/publications/i/item/9789240051683 (accessed March 29, 2023).

[12] World Health Organization. Availability, safety and quality of blood products (2010). Available online at: https://www.who.int/publications/i/item/WHA63.12 (accessed March 29, 2023).

[13] World Health Organization. Guidance on centralization of blood donation testing and processing (2021). Available online at: https://apps.who.int/iris/handle/10665/340182

[14] World Health Organization. Who Global Benchmarking Tool Plus (GBT+) For Evaluation of National Regulatory System of Medical Products Vigilance (VL): Indicators and Fact Sheets. (2019). Geneva: World Health Organization.

[15] Khadem Broojerdi A Baran Sillo H Ostad Ali Dehaghi R Ward M Refaat M Parry J. The world health organization global benchmarking tool an instrument to strengthen medical products regulation and promote universal health coverage. Front Med. (2020) 7:457. 10.3389/fmed.2020.00457 32974367 PMC7466745

[16] Guzman J O'Connell E Kikule K Hafner T. The WHO global benchmarking tool: a game changer for strengthening national regulatory capacity. BMJ Global Health. (2020) 5e003181. 10.1136/bmjgh-2020-003181 32784212 PMC7418656

[17] Shi J Chen X Hu H Ung COL. Benchmarking drug regulatory systems for capacity building: an integrative review of tools, practice, and recommendations. Int J Health Policy Manag. (2023) 12:8100. 10.34172/ijhpm.2023.8100 38618782 PMC10699822

[18] Rägo L Sillo H 'T Hoen E Zweygarth M. Regulatory framework for access to safe, effective quality medicines. Antivir Ther. (2014) Suppl 3:69–77. 10.3851/IMP2902 25310085

[19] Roth L Bempong D Babigumira JB Banoo S Cooke E Jeffreys D. Expanding global access to essential medicines: investment priorities for sustainably strengthening medical product regulatory systems. Global Health. (2018) 14:102. 10.1186/s12992-018-0421-2 30382856 PMC6211488

[20] World Health Organisation. Global Benchmarking Tool (GBT) for evaluation of national regulatory system of medical products Manual for benchmarking and formulation of institutional development plans. Geneva (2023).

[21] Burnouf T. Blood products: unmet needs for essential medicines. Lancet Haematol. (2019) 6:e598–9. 10.1016/S2352-3026(19)30217-0 31631024

[22] Seitz R Heiden M Nübling CM Unger G Löwer J. The harmonization of the regulation of blood products: a European perspective. Vox Sang. (2008) 94:267–76. 10.1111/j.1423-0410.2007.01026.x 18179678

[23] Teleconference B. European commission directorate-general for health and food safety. Directorate B-Health systems, medical products and innovation B4-Medical products: quality, safety, innovation stakeholder workshop with blood competent authorities substances of human origin expert group (CASoHO E01718) WORKSHOP TOPIC: Regulating for sufficiency-blood and plasma summary minutes (2021).

[24] European Parliament. Directive 2002/98/EC of the European Parliament and of the Council of 27 January 2003 setting standards of quality and safety for the collection, testing, processing, storage and distribution of human blood and blood components. Official Journal of the European Union, L 033 (2003). p. 30–40. Available online at: https://eur-lex.europa.eu/legal-content/EN/TXT/?uri=celex%3A32002L0098 (accessed April 28, 2023).

[25] European Medicines Agency. Plasma master file (PMF) certification | European Medicines Agency. Available online at: https://www.ema.europa.eu/en/human-regulatory/overview/plasma-master-file-pmf-certification (accessed April 27, 2023).

[26] European Parliament. Commission Directive 2004/33/EC of 22 March 2004 implementing Directive 2002/98/EC of the European parliament and of the council as regards certain technical requirements for blood and blood components. Official Journal of the European Union, L0033 (2004). p. 29–35. Available online at: https://eur-lex.europa.eu/legal-content/EN/TXT/?uri=CELEX:32004L0033 (accessed May 21, 2024).

[27] European Parliament. Commission Directive 2005/62/EC of 30 September 2005 implementing Directive 2002/98/EC of the European parliament and of the council as regards community standards and specifications relating to a quality system for blood establishments. Official Journal of the European Union, L 256 (2005). p. 41–8. Available online at: https://eur-lex.europa.eu/legal-content/EN/TXT/?uri=OJ:L:2005:256:TOC (accessed May 21, 2024).

[28] Wood EM Ang AL Bisht A Bolton-Maggs PH Bokhorst AG Flesland O. International haemovigilance: what have we learned and what do we need to do next? Transfus Medicine. (2019) 29:221–30. 10.1111/tme.12582 30729612

[29] World Health Organization. List of National Regulatory Authorities (NRAs) operating at maturity level 3 (ML3) 1 and maturity level 4 (ML4) 2 (as benchmarked against WHO Global Benchmarking Tool (GBT). Geneva: World Health Organization (2023). Available online at: https://www.who.int/publications/m/item/list-of-nras-operating-at-ml3-and-ml4 (accessed October 1, 2024).

[30] World Health Organization. WHO Action framework to advance universal access to safe, effective and quality assured blood products (2020).

[31] Harris Rachelle LR Poon Charlotte Raj Nimisha Barron Anthony. Strengthening regulatory systems in LMICs: improving the sustainability of the vaccine innovation ecosystem in Africa. (2022). p. 113.

[32] World Health Organization. Guidelines on Management of Blood and Blood Components as Essential Medicines. Geneva: World Health Organization (2017).

[33] Abdurrahman G Samukange W Lyoko N Shonhiwa NO Kafere C Nübling CM. Regulation of blood-screening in vitro diagnostics in sub-Saharan African countries remains a challenge. Front Med. (2023) 10:1252721. 10.3389/fmed.2023.1252721 37854664 PMC10579816

[34] Samukange WT Klümpers V Kafere C Schuler F Tirane FJ Kabaso B. Report from the GHPP-BloodTrain inspection workshop in Harare the 20th to the 24th of May 2019. Biologicals. (2020) 68:125–8. 10.1016/j.biologicals.2020.08.012 32907761

[35] Atemnkeng J Heinrich K Samukange WT Kafere C Mbunkah HA Schoner C. Report on the GHPP-BloodTrain online workshop on “Authorisation and licensing of blood establishments - review of quality documentation of blood and blood components”, 5th-8th of July 2021. Biologicals. (2022) 80:1–5. 10.1016/j.biologicals.2022.10.003 36328931

[36] Mbunkah HA Kafere C Samukange W Nübling M Reinhardt J. Congress report: online workshop on assessment of technical files for blood screening in vitro diagnostics for sub-Sahara African countries. Transfus Med. (2022) 32:467–74. 10.1111/tme.12925 36264545 PMC10091766

[37] Chaw EE Suntornsuk L. Regulatory landscape analysis of Myanmar food and drug administration based on the world health organization global benchmarking tool. Pharm Sci Asia. (2022) 49:498–505. 10.29090/psa.2022.05.22.174

[38] Keyter A Gouws J Salek S Walker S. The regulatory review process in South Africa: challenges and opportunities for a new improved system. Ther Innov Regul Sci. (2018) 52:449–58. 10.1177/2168479018776649 29848046 PMC6047299

[39] Sithole T Mahlangu G Salek S Walker S. Evaluation of the regulatory review process in Zimbabwe: challenges and opportunities. Ther Innov Regul Sci. (2021) 55:474–89. 10.1007/s43441-020-00242-z 33387356 PMC8021537

[40] Haldane V De Foo C Abdalla SM Jung AS Tan M Wu S. Health systems resilience in managing the COVID-19 pandemic: lessons from 28 countries. Nat Med. (2021) 27:964–80. 10.1038/s41591-021-01381-y 34002090

[41] Shabani JBB Kayitare E Nyirimigabo E Habyalimana V Murindahabi MM Ntirenganya L. The capacity of young national medicine regulatory authorities to ensure the quality of medicines: case of Rwanda. J Pharm Policy Pract. (2022) 15:90. 10.1186/s40545-022-00492-2 36434730 PMC9700871

[42] Sani NM McAuslane N Kasbon SH Ahmad R Yusof FAM Patel P. An evaluation of Malaysian regulatory process for new active substances approved in 2017 using the OpERA methodology. Ther Innov Regul Sci. (2020) 54:1215–24. 10.1007/s43441-020-00140-4 32865804 PMC7458937

[43] Scharifi O Bihler T. European Union's twinning instrument for capacity building in EU neighbour administrations a case of multi-level-governance? PSG XIV EU administration and multi-level governance work in progress. EGPA 39th Annual Conference 2017. Milan, Italy (2017).

[44] Roch S. Possibilities and limits of cross-country administrative cooperation at Europe's Fringes: a process perspective on EU twinning in Moldova and Lebanon (2016).

[45] Ndomondo-Sigonda M Azatyan S Doerr P Agaba C Harper KN. Best practices in the African medicines regulatory harmonization initiative: perspectives of regulators and medicines manufacturers. PLoS Global Public Health. (2023) 3:e0001651. 10.1371/journal.pgph.0001651 37186241 PMC10132525

[46] Ngum N Ndomondo-Sigonda M Walker S Salek S. Regional regulatory harmonisation initiatives: their potential contribution to the newly established African medicines agency. Regul Toxicol Pharmacol. (2023) 145:105497. 10.1016/j.yrtph.2023.105497 37778434

[47] Mashingia J Ngum N Ndomondo-Sigonda M Kermad A Bujar M Salek S. Regulatory performance of the East African community joint assessment procedure: the way forward for regulatory systems strengthening. Regul Toxicol Pharmacol. (2023) 140:105383. 10.1016/j.yrtph.2023.105383 36933643

