# Supplementary material for: Bridging the gap: enhancing blood regulatory functions in African contexts through comparative analysis
Source: Front Med (Lausanne). 2025 May 1;12:1519719. doi: 10.3389/fmed.2025.1519719 (PMC12078219; doi:10.3389/fmed.2025.1519719)
Supplement: Supplementary file 1 [file Table_1.docx]

Bridging the Gap: Enhancing Blood Regulatory Functions in African contexts through Comparative Analysis

Supplementary Table 1a: Registration and marketing authorisation (plasma – derived medicines) function indicators and sub-indicators in the WHO GBT+ Blood (ML1 – ML3)

| **Indicator** | **Sub-indicator** | **Maturity Level** |
| --- | --- | --- |
| MA01 Legal provisions, regulations and guidelines required to define regulatory framework of registration and/or marketing authorization. | MA01.01: There are legal provisions that require the receipt of a registration or marketing authorization (MA) before placing the product on the market. | **1** |
| MA01 Legal provisions, regulations and guidelines required to define regulatory framework of registration and/or marketing authorization. | MA01.02: There are legal provisions that require the NRA to withhold, suspend, withdraw or cancel an MA if there are concerns regarding quality, safety or efficacy issues. | **1** |
| MA01 Legal provisions, regulations and guidelines required to define regulatory framework of registration and/or marketing authorization. | MA01.03: There are legal provisions that require demonstration of the product quality, safety and efficacy prior to registration or MA | **1** |
| MA01 Legal provisions, regulations and guidelines required to define regulatory framework of registration and/or marketing authorization. | MA01.04: There are legal provisions or regulations limiting the duration of the validity of the MA and requiring periodic reviews of MAs (i.e. renewals) | **2** |
| MA01 Legal provisions, regulations and guidelines required to define regulatory framework of registration and/or marketing authorization. | MA01.05: There are regulations or guidelines for the definitions, types and the scope of variations along with the required documentation for these variations | **3** |
| MA01 Legal provisions, regulations and guidelines required to define regulatory framework of registration and/or marketing authorization. | MA01.06: There are legal provisions to cover circumstances under which the routine MA procedures may not be followed (e.g., for public health interest) | **1** |
| MA01 Legal provisions, regulations and guidelines required to define regulatory framework of registration and/or marketing authorization. | MA01.07: There are legal provisions or regulations that define regulatory requirements to approve donation of medical products. | **1** |
| MA01 Legal provisions, regulations and guidelines required to define regulatory framework of registration and/or marketing authorization. | MA01.08: Legal provisions or regulations allow the NRA to recognize and/or rely on MA-relevant decisions, reports or information from other NRAs or regional and international bodies | **1** |
| MA01 Legal provisions, regulations and guidelines required to define regulatory framework of registration and/or marketing authorization. | MA01.09: Specific guidelines on the quality, nonclinical and clinical aspects are established and implemented | **3** |
| MA01 Legal provisions, regulations and guidelines required to define regulatory framework of registration and/or marketing authorization. | MA01.10: There are guidelines on the format and content for submission of MA applications that are consistent with the WHO or other internationally accepted standards | **3** |
| MA01 Legal provisions, regulations and guidelines required to define regulatory framework of registration and/or marketing authorization. | MA01.11: There are guidelines for MA holders that define the types and scope of variations, the format and content to be used for documenting the variations, and the identification of those variations that require prior approval or notification. | **3** |
| MA01 Legal provisions, regulations and guidelines required to define regulatory framework of registration and/or marketing authorization. | MA01.12: There are established guidelines that cover circumstances under which the routine MA procedures may not be followed (e.g., for public- health interest) | **3** |
| MA01 Legal provisions, regulations and guidelines required to define regulatory framework of registration and/or marketing authorization. | MA01.13: There are guidelines on the content of product information leaflets, SPC-like information, and product packaging and labelling. | **3** |
| MA02 Arrangement for effective organization and good governance. | MA02.01: There is a defined structure with clear responsibilities to conduct registration or MA activities | **2** |
| MA02 Arrangement for effective organization and good governance. | MA02.02: Documented and implemented procedures exist to ensure involvement and communication with all relevant regulatory entities as necessary | **3** |
| MA03 Human resources to perform registration and marketing authorization activities. | MA03.01: Sufficient competent staff (i.e., education, training, skills and experience) are assigned to perform MA or registration activities | **3** |
| MA03 Human resources to perform registration and marketing authorization activities. | MA03.02: Duties, functions, and responsibilities of the staff in charge of MA or registration activities are established and updated in the respective job descriptions. | **3** |
| MA03 Human resources to perform registration and marketing authorization activities. | MA03.03: Training plan developed, implemented and updated at least once a year for staff in charge of MA or registration activities. | **3** |
| MA03 Human resources to perform registration and marketing authorization activities. | MA03.04: The NRA generates and maintains records of staff training activities and training effectiveness verification. | **3** |
| MA04 Procedures established and implemented to perform registration and/or marketing authorization | MA04.01: Documented procedures and tools are implemented for the assessment of the different parts of the application (i.e., quality, and efficacy) and for the assessment of specific requirements applicable to specific classes of medical products | **3** |
| MA04 Procedures established and implemented to perform registration and/or marketing authorization | MA04.02: Documented procedures have been implemented to renew and/or to periodically review the MAs granted | **3** |
| MA04 Procedures established and implemented to perform registration and/or marketing authorization | MA04.03: Documented procedures are implemented for assessing applications for variations of MAs. | **3** |
| MA04 Procedures established and implemented to perform registration and/or marketing authorization | MA04.04: The same criteria apply for assessing applications regardless of the origin of or destination for the medical products (e.g., domestic, foreign, public sector, or private sector) | **3** |
| MA04 Procedures established and implemented to perform registration and/or marketing authorization | MA04.06: Timelines for the assessment of the applications are defined and an internal tracking system has been established to monitor adherence to the targeted time frames | **3** |
| MA04 Procedures established and implemented to perform registration and/or marketing authorization | MA04.07: There are documented mechanisms to handle non-routine registration or MA requirements in special situations (e.g., public-health interest) | **3** |
| MA04 Procedures established and implemented to perform registration and/or marketing authorization | MA04.08: SPC-like, labelling and packaging information are approved by the NRA as part of the MA procedure | **3** |
| MA04 Procedures established and implemented to perform registration and/or marketing authorization | MA04.09: GMP inspection report and/or certification is considered as part of the MA process. | **3** |
| MA04 Procedures established and implemented to perform registration and/or marketing authorization | MA04.10: The regulations and guidelines for good review practices (GRevPs) are developed or recognized and implemented. | **3** |
| MA04 Procedures established and implemented to perform registration and/or marketing authorization | *MA04.12 There is a requirement for the applicant to include a list of all the blood and plasma collection establishments that collected the plasma used in the product | **2** |
| MA04 Procedures established and implemented to perform registration and/or marketing authorization | *MA04.13 Specifications related to the quality and safety of plasma for fractionation are defined and under the supervision of the NRA | **2** |
| MA04 Procedures established and implemented to perform registration and/or marketing authorization | *MA04.14 Selection, deferral and transmissible-disease testing requirements for plasma donors are established. | **2** |
| MA05 Mechanism exists to promote transparency, accountability and communication. | MA05.01: Web site or other official publication with SPC-like information is available and regularly updated | **3** |
| MA05 Mechanism exists to promote transparency, accountability and communication. | MA05.02: Updated list of all medical products granted MA is regularly published and publicly available | **3** |
| MA06 Mechanism in place to monitor regulatory performance and output | MA06.01: There is a database of all product applications received, approved, rejected, suspended or withdrawn along with their supporting documentation. | **3** |

*specific blood and blood product related sub-indicators

Supplementary Table 1b: Approval of blood and blood components including plasma for fractionation or process function indicators and sub-indicators in the WHO GBT+ Blood (ML1 – ML3)

| **Indicator** | **Sub-indicator** | **Maturity Level** |
| --- | --- | --- |
| AB01 Legal provisions exist for a system to ensure quality, safety and efficacy of blood and blood components. | AB01.01 An approval system is required that includes any imported products. | **1** |
| AB01 Legal provisions exist for a system to ensure quality, safety and efficacy of blood and blood components. | AB01.02 The NRA has the authority to issue an approval, to suspend an approval and to withdraw an approval if the product or process is considered unsafe or does not comply with regulatory requirements. | **1** |
| AB02 A system for ensuring quality, safety and efficacy of blood and blood components is established and operational. | AB02.02 Specifications related to quality, safety and efficacy of blood and blood components are defined and under the supervision of the NRA. | **2** |
| AB02 A system for ensuring quality, safety and efficacy of blood and blood components is established and operational. | AB02.03 The critical standards for product manufacturing are legally binding and include donor selection, laboratory testing, component preparation, storage, issuance, tracking, tracing, record keeping, and safe disposal of units not meeting specifications. | **2** |
| AB02 A system for ensuring quality, safety and efficacy of blood and blood components is established and operational. | AB02.04 Legal authority to make exceptions is clearly defined. | **2** |
| AB02 A system for ensuring quality, safety and efficacy of blood and blood components is established and operational. | AB02.05 Requirements and standards are aligned with WHO or other internationally recognized standards. | **2** |
| AB02 A system for ensuring quality, safety and efficacy of blood and blood components is established and operational. | AB02.06 Plasma for fractionation meets WHO or other internationally recognized standards. | **3** |
| AB03 Donor selection and deferral criteria are established as appropriate for the intended use of the component. | AB03.01 Donor selection and deferral criteria (i.e., temporary and permanent deferrals) are consistent with current science and take into account the health of the donor and the safety and suitability of the donation. | **1** |
| AB03 Donor selection and deferral criteria are established as appropriate for the intended use of the component. | AB03.02 Mechanisms for the regular review and updating of donor selection and deferral criteria are in place and take into consideration issues that might have a negative impact on the quality and safety of blood and blood components (e.g., epidemiological situation or emerging diseases). | **3** |
| AB04 Transmissible-disease testing requirements are established and are appropriate for the intended use of the component. | AB04.01 Mechanisms are in place for regularly reviewing (e.g., by qualified experts in epidemiology) and updating of the test requirements. | **3** |
| AB04 Transmissible-disease testing requirements are established and are appropriate for the intended use of the component. | AB04.02 Epidemiological data regarding the prevalence and incidence of infectious disease markers in blood donors are available and regularly updated. | **3** |
| AB05 Labelling requirements are established. | AB05.01 Each blood component has a unique and clear identifier and is fully traceable | **1** |
| AB05 Labelling requirements are established. | AB05.02 Original labelling and significant amendments are submitted to the NRA and assessed by the NRA prior to implementation | **1** |
| AB05 Labelling requirements are established. | AB05.03 Product labelling includes information on the risks and benefits of product use | **3** |
| AB05 Labelling requirements are established. | AB05.04 Product labelling requirements are aligned with WHO or other internationally recognized standards. | **3** |
| AB06 An approval system for blood and blood components is operational | AB06.01 Mechanisms for assessment exist and include relevant aspects of quality, safety and efficacy (when applicable) of blood and blood components including plasma for fractionation. | 3 |
| AB06 An approval system for blood and blood components is operational | AB06.02 Guidelines exist that specify the content and format of the application for approval and the procedures to be followed when submitting an application | 3 |
| AB06 An approval system for blood and blood components is operational | AB06.03 Written guidelines for assessment of applications are implemented | 3 |
| AB06 An approval system for blood and blood components is operational | AB06.04 Appeal procedures are in place | 3 |
| AB06 An approval system for blood and blood components is operational | AB06.05 Assessment reports are prepared and used as a reference for decision-making | 3 |
| AB07: There is a requirement that post-approval changes in product preparation be submitted to and assessed by the regulatory authority. | AB07.01: Written guidelines for applicants are available that define the types and scope of post-approval changes to product preparation for which submission of documentation is required. | 3 |
| AB07: There is a requirement that post-approval changes in product preparation be submitted to and assessed by the regulatory authority. | AB07.02: Written procedures for assessment exist and are based on the type of changes (e.g., significant, notifiable, and administrative). | 3 |
| AB08 Appropriate assessment expertise is available. | AB08.01. Access to experts (internal or external) with relevant qualifications and experience for assessment of blood and blood components (i.e., preclinical, clinical and quality data) is assured | 3 |
| AB08 Appropriate assessment expertise is available. | AB08.02 Written procedures are in place for selection, management, and use of external experts | 3 |
